# Influenza Viruses and Vaccines: The Role of Vaccine Effectiveness Studies for Evaluation of the Benefits of Influenza Vaccines

**DOI:** 10.3390/vaccines10050714

**Published:** 2022-05-01

**Authors:** Claudia Maria Trombetta, Otfried Kistner, Emanuele Montomoli, Simonetta Viviani, Serena Marchi

**Affiliations:** 1 Department of Molecular and Developmental Medicine, University of Siena, 53100 Siena, Italy; emanuele.montomoli@unisi.it (E.M.); simonetta.viviani@unisi.it (S.V.); serena.marchi2@unisi.it (S.M.); 2 VisMederi srl, 53100 Siena, Italy; kistner@vismederi.com; 3 VisMederi Research srl, 53100 Siena, Italy

**Keywords:** influenza virus, vaccine, effectiveness

## Abstract

Influenza is a vaccine preventable disease and vaccination remains the most effective method of controlling the morbidity and mortality of seasonal influenza, especially with respect to risk groups. To date, three types of influenza vaccines have been licensed: inactivated, live-attenuated, and recombinant haemagglutinin vaccines. Effectiveness studies allow an assessment of the positive effects of influenza vaccines in the field. The effectiveness of current influenza is suboptimal, being estimated as 40% to 60% when the vaccines strains are antigenically well-matched with the circulating viruses. This review focuses on influenza viruses and vaccines and the role of vaccine effectiveness studies for evaluating the benefits of influenza vaccines. Overall, influenza vaccines are effective against morbidity and mortality in all age and risk groups, especially in young children and older adults. However, the effectiveness is dependent on several factors such as the age of vaccinees, the match between the strain included in the vaccine composition and the circulating virus, egg-adaptations occurring during the production process, and the subject’s history of previous vaccination.

## 1. Introduction

Influenza disease, usually called “the flu”, is a contagious respiratory illness caused by influenza viruses. The common symptoms are fever, aches, chills, chest discomfort, cough, and headache [1]. The incubation period is very short, typically from 1 to 4 days [2]. While the majority of infected subjects recover, some develop complications, particularly at-risk groups such as pregnant women, young children, the elderly, and individuals with chronic medical conditions [1,3]. One of the most common influenza-related complications is a secondary bacterial infection by *Streptococcus pneumoniae*, which increases the morbidity and mortality of influenza infection [4,5].

The World Health Organization (WHO) estimates that influenza alone results in 290,000–650,000 deaths each year due to respiratory diseases without taking into account deaths from other potentially influenza-related diseases [6]. Before the COVID-19 pandemic, Italy was experiencing peaks in influenza-related death rates, especially among the elderly during some winter seasons. An Italian study conducted over the influenza seasons 2013–2014 to 2016–2017 reported a high proportion of deaths among the elderly (seasons 2014–2015 and 2016–2017) and also high rates in children between 0 and 4 years [7]. Vaccination is still the most effective way of preventing and controlling influenza morbidity and mortality and as a consequence the WHO recommends 75% vaccine coverage for the vulnerable risk group of elderly persons [8]. As reported by the Italian Ministry of Health, in the 2020–2021 influenza season, vaccination coverage among subjects aged 65 years and older was 65.3%, higher than the previous years but still not optimal [9].

Influenza viruses spread mainly from person to person through droplets generated by sneezing, coughing and talking [2]. While influenza viruses are globally detected all year round, in temperate climates influenza epidemics occur during winter: November–March in the Northern hemisphere and May–September in the Southern hemisphere [2,10,11,12]. By contrast, influenza seasonality is less defined in tropical regions [13]. Although the seasonality of influenza in temperate regions has been well characterized, the factors influencing seasonality are still under study. Crowding, cold weather, reduced solar radiation, and low indoor humidity are usually related to influenza epidemics. In addition, intrinsic host factors, which could impact the immune system such as melatonin linked to light/dark cycles and vitamin D, may play a role in influenza seasonality [12,13,14].

Influenza viruses are enveloped viruses belonging to the *Orthomyxoviridae* family. There are four genera (A, B, C, and D), which are classified on the basis of antigenic differences in the nucleoprotein and matrix protein [15]. Influenza A viruses (IAVs) infect avian and mammalian species with swine and poultry constituting the two key reservoirs. Influenza B viruses (IBVs) are exclusive to humans, apart from seals. Influenza C viruses (ICVs) are mainly human pathogens and are responsible for lower respiratory tract infections, especially during childhood. However, they have also been detected in pigs, dogs, and cattle. The most recent virus is the influenza D virus (IDV), which was first isolated in 2011 from a swine with respiratory disease. However, both epidemiological and serological studies support the hypothesis that cattle are the natural reservoir. To date there is no direct evidence of IDV disease in humans, but antibodies against IDV have been detected, underlining the potential ability of this virus to infect and elicit an immune response in humans and especially in people working with animals and/or exposed to cattle [15,16,17,18,19,20].

IAVs are further classified in subtypes on the basis of the combinations of the two main surface glycoproteins: haemagglutinin (HA) and neuraminidase (NA). To date, 18 HAs and 11 NAs have been identified. HA constitutes roughly 80% of the virus surface and plays a key role in the attachment and entry of the virus into the host cell. The antibody response to influenza virus glycoproteins is mostly directed against HA. On the basis of the HA expressed, influenza viruses are further classified into two phylogenetic groups: group 1 and group 2 [21]. NA is responsible for release of the viral progeny from an infected cell. NA antibodies are not able to prevent infection but may contribute to mitigating the severity and duration of the disease [15,22,23,24].

IBVs are divided into two antigenic lineages (B/Victoria and B/Yamagata), which diverged in the 1970s and have co-circulated globally in humans since 1985 [25]. The other influenza viruses currently circulating in humans are type A, subtypes H1N1 and H3N2.

Influenza A and B viruses are subject to frequent mutations of their glycoprotein sequences—mainly those of HA and, at a slower rate, those of the NA—owing to the lack of proofreading polymerase activities [22,26]. “Antigenic drift” refers to minor antigenic changes that enable the virus to escape the host’s immune response and is responsible for annual influenza epidemics. Antigenic drift can sometimes involve changes in the glycosylation patterns of viral glycoproteins. The H3N2 viruses evolve particularly rapidly and more unpredictably than other seasonal influenza viruses. “Antigenic shift”, which occurs only in IAVs, consists of abrupt, major changes in HA or HA/NA, resulting in new influenza subtypes unknown to the human immune system resulting in the lack of immunological protection; the new subtype therefore may have pandemic potential. Antigenic shift may be the result of direct mutational changes of a zoonotic influenza enabling the virus to infect humans directly and to support efficient human-to-human transmission. Alternatively, it can occur through reassortment between zoonotic influenza viruses and seasonal human influenza viruses. The last one means that two influenza viruses infect a common host, such as a pig, resulting in a new influenza virus that has some antigenic determinants of one virus and the host tropism and pathogenicity of the other [15,24,27]. Influenza pandemics occur approximately every 10 to 40 years. However, as we have learned from the COVID-19 pandemic, it is still not possible to predict when, where, and how severely they will strike [15,28]. The first pandemic of the XXI century was caused by a new influenza A/H1N1 virus of swine origin, which appeared in Mexico in March and early April 2009 which substituted the former human seasonal influenza H1N1 virus. This pandemic was relatively mild and affected children and young and middle-aged adults more severely than other groups. This unusual pattern of age-related morbidity and mortality was due to pre-existing cross-protection against the new H1N1 virus in subjects older than 60 years. The most devastating pandemic in human history was the so-called “Spanish Flu” in 1918–1919, which caused almost 50 million deaths worldwide and was dubbed “the mother of all pandemics”. Since that time, all influenza A pandemics have been caused by descendants of the 1918 virus [29,30,31,32,33]. The other two great pandemics were the 1957 “Asian Flu”, caused by an H2N2 virus, and the 1968 “Hong Kong Flu”, caused by an H3N2 virus that replaced the H2N2 and which still circulates worldwide as seasonal IAV [34,35,36].

Pandemics are quite difficult to predict. A key step in pandemic preparedness is the prompt detection of novel influenza strains as they emerge and before being efficiently transmitted among humans. Some influenza viruses are of particular concern such as avian viruses, although they have not yet caused a pandemic. Since the first outbreak of avian influenza in Hong Kong in 1997, the WHO has reported a total of 239 cases of human infection with A/H5N1 virus, as of 3 February 2022, with 134 deaths (case fatality rate: 56%) [37]. In addition to H5N1, H7N9, and H10N8, other avian viruses, such as H10N3, are potential pandemic candidates. This highlights the importance of surveillance in both humans and animals, such as wild birds and poultry, which is still the key to controlling the emergence of novel avian influenza strains [38,39]. Notably, at the beginning of 2022, no new cases of human infection by the H7N9 avian influenza virus were reported in the Western Pacific Region. Since early 2013, laboratory-confirmed human infections have totaled 1568, with 616 deaths [40]. Of 1568 cases, mutations in the HA gene have been reported in 33 cases, providing evidence of a change to pathogenicity in poultry. As yet, however, there is no evidence of sustained human-to-human transmission, and human infections are unusual. Nevertheless, careful monitoring is crucial in order to promptly identify viral changes and transmission patterns that could make the virus a threat to humans [34,41,42,43,44].

## 2. Annual Update of Seasonal Influenza Vaccines

Owing to the evolving nature of influenza viruses, the composition of vaccines needs annual evaluation in order to include the seasonal viruses predicted to circulate during the next influenza season. Influenza vaccines may be trivalent, containing the A/H1N1 virus, A/H3N2 virus, and one lineage of the B virus, or quadrivalent, containing both B lineages. Since the 2013–2014 influenza season in the Northern Hemisphere, the WHO has recommended the inclusion of both B lineages, as this is deemed to provide broader protection against IBVs. Indeed, the inclusion of one B lineage confers limited protection against the other lineage. Moreover, in some influenza seasons (such as 2001–2002 and 2010–2011) the predominant circulating lineage was different from the one included in the vaccine, resulting in a limited vaccine effectiveness (VE) [41,45,46,47]. Since 1998, the WHO has held twice-yearly consultations with vaccine regulatory agencies, vaccine manufacturers, members of the WHO Global Influenza Surveillance and Response System (GISRS), global public health laboratories, and academia: one in February for the Northern hemisphere and one in September for the Southern hemisphere. The WHO recommendations provide a guide to which influenza viruses should be included in influenza vaccines for use in the next influenza season. However, it is the responsibility of each national regulatory authority to approve or modify the vaccine composition [48,49,50]. The GISRS network, a global system of public health institutions, is coordinated by the WHO, and currently consists of 148 National Influenza Centres (NICs), 7 WHO Collaborating Centres (CCs) for Influenza, 4 WHO Essential Regulatory Laboratories (ERLs), and 13 H5 Reference Laboratories. The GISRS plays a key role in global influenza risk assessment and performs year-round virological surveillance. It has been estimated that the GISRS processed an average of 3.4 million specimens every year from 2014 to 2019, a figure that increased to 6.7 million in 2020 and 2021. The WHO recommendations of viruses for inclusion in the annual seasonal vaccines are based on GISRS surveillance [48].

Vaccine production takes 6 to 8 months from the WHO recommendations. This is a tight schedule and a complex process with limited flexibility and requires compromises. Consequently, vaccine manufacturers sometimes prefer to start the production of at least one antigen before the official recommendation is issued, in order to be able to manage unexpected events. However, the current system does not allow manufacturers to anticipate the WHO recommendations; thus, in the event of mismatch in April/May, there is not enough time to thoroughly investigate the viral variant that has emerged and, if necessary, to prepare a well-matched vaccine. This could impact on the VE, as happened during the 2014–2015 season in the Northern hemisphere, when an antigenic variation of the H3N2 virus emerged and only little or no VE was observed [24,46,50,51].

However, other crucial factors contribute to the timely production, evaluation, and delivery of vaccines before the next influenza season. First, the preparation of the Candidate Vaccine Virus (CVV). The CVV is a virus prepared by the CCs for potential use in vaccine manufacturing and should be antigenically similar to the virus recommended in the next influenza season. Since the majority of vaccines are still produced in eggs, the CVV needs to replicate well in eggs. CVVs are crucial for the timely production of egg-based vaccines. In the case of cell-based manufacturing, a different CVV is prepared, while influenza vaccines based on recombinant DNA technology do not require CVVs as they are based on the genetic information of the recommended vaccine viruses [3,52].

Once the CVVs have been ascertained to be antigenically related to the recommended vaccine strains (defined as “-like”), they are supplied to manufacturers, who generate “seed viruses” for the production of inactivated vaccines. A key step of the production process is the availability of “high-yield” (hy) CVV. CVV wildtype viruses poorly grown in eggs will extend the time to produce a sufficient amount of vaccine antigen. The hy, or high-growth reassortant (hgr), was proposed by Kilbourne in 1969 and developed by co-infection of A/PR/8/34 (PR8), a hy donor virus, along with the recommended wild type “target” virus. The generation of hy reassortant vaccine seed viruses is the result of combining the HA and NA from the wild type “target” virus with 1–6 of the remaining genes from the hy donor virus PR8 [50,51].

Other factors which can impair, or delay vaccine production include the timely production of reagents for determination of the HA antigen content in the vaccine and ferret antisera for confirmation of antigenic identity [46,50].

The WHO GISRS, in collaboration with animal health partners, also performs surveillance for zoonotic events, and decisions are made at least twice a year regarding the need to develop CVVs for the purpose of pandemic preparedness [53]. Over the last 20 years, several zoonotic influenza events have been detected. The selection and development of zoonotic CVVs are aimed at maintaining a bank of viruses that can be promptly used for vaccine development and also at aiding manufacturers that want to develop pilot lots of vaccines [41,52].

## 3. Licensed Seasonal Influenza Vaccines

The degree of protection conferred by vaccination depends on a complex interplay between vaccine composition and circulating influenza viruses, the age of vaccinees and their history of previous exposure to influenza viruses, and/or influenza vaccinations, as well as product-specific factors such as the vaccine formulation.

To date, three types of influenza vaccines have been licensed: inactivated, live-attenuated, and recombinant HA vaccines. The advantages and disadvantages of licensed seasonal influenza vaccines are summarized in Table 1.

The inactivated influenza vaccines (IIVs) are the most widely used ones and can be whole virus, split virus, or subunit vaccines; they are administered by intramuscular or subcutaneous injection. Split and subunit IIVs can be administered to all age groups from six months onwards. Whole virus vaccines have been extensively used in humans but are no longer in use in most parts of the world owing to their relatively high reactogenicity. Subunit or split virus vaccines are the most widespread [15,23,46,54]. The latter are prepared by disrupting the viral membrane chemically. Subunit vaccines contain only the HA and NA glycoproteins of the virus. Currently, none of the licensed vaccines are adjuvanted, with the exception of an MF-59 adjuvanted subunit vaccine licensed for persons of 65 years of age and older to overcome the weakened immune response in this age group, also known as immunosenescence [55]. Another vaccine licensed for people of 60 (Europe) or 65 (USA) years of age and older is high-dose IIV, which contain a four times higher amount of the HA antigen than the standard dose [56,57].

IIVs are mainly produced in embryonated hens’ eggs, harvested from the allantoic cavity and manufactured according to the type of vaccine. Egg-based production is a long- and well-established traditional system. Its main advantages are the availability of extensive safety data, cost-effectiveness, and high yields of influenza virus antigens. On the other hand, it has several limitations, which have prompted the development and implementation of other platforms. One major issue is the need for a huge number of embryonated hens’ eggs; one or two eggs are usually required for one dose of vaccine. Moreover, subjects with an egg allergy are not recommended to receive this kind of vaccine, owing to the theoretical risk of an anaphylactic reaction, since small amounts of egg proteins may be present in the vaccine. Finally, some viruses, such as H3N2, grow poorly in eggs and could undergo some egg-adaptive changes that could reduce the VE [46,54]. The current alternative for vaccine production is the cell-culture platform. The main advantage of this platform is its independence from the supply of eggs, a crucial aspect in the event of an avian pandemic, as the cells can be cryo-conserved and used at any time when needed, in contrast to the supply of eggs which needs a diligent advance planning for at least 6 months. Another advantage is the freedom of the vaccine from any egg component. Another important advantage of the cell culture platform is in overcoming the risk of egg-adapted mutations which can affect the antigenicity of vaccine viruses. A cell-based quadrivalent IIV produced by Seqirus has been approved and is now widely available [57,58]. However, this production system also has some disadvantages such as shorter experience, the need to qualify production facilities, an extended quality control program including testing for adventitious agents, and higher production costs [15,46]. All current IIVs are standardized with regard to HA content, but not to NA content [59].

Live influenza vaccines (LAIVs) are administered intranasally, thereby miming natural infection and inducing broader humoral and cellular immune responses than IIVs, including additional mucosal IgA responses in the upper respiratory tract. These attenuated viruses are cold-adapted and are able to replicate at 25 °C, the temperature of the nasal passage, but not at a temperature higher than 35 °C, that of the respiratory tract. LAIVs can provide protection against both well-matched and non-matching influenza strains. These vaccines are administered to healthy subjects, aged 2–49 years, according to the country-specific regulations; however, the use of live viruses makes these vaccines unsuitable for immunocompromised subjects [15,46].

The first vaccine produced by a modern recombinant DNA technology was Flublok^®^, which was developed by Protein Science, Meriden, CT, and acquired by Sanofi Pasteur. Initially approved by the Food and Drug Administration in 2013 for use in adults aged 18–49 years, the quadrivalent version replaced the previous one in 2017 for use in adults aged 18 years and older. The quadrivalent vaccine, named Supemtek^®^, was also authorized by the European Medicines Agency in 2020 [60,61,62]. This technology offers several important advantages; indeed, it does not require eggs or CVVs, and the manufacture of the HA of the target virus starts from the viral RNA sequence by in vitro synthesis of the corresponding DNA. This DNA is cloned in a baculovirus, preferably *Autographa californica* multiple-capsid nuclear polyhedrosis virus [63,64,65]. This approach may be particularly important, allowing a prompt response in the event of a pandemic, based on sequence information rather than the availability of an infectious virus isolate. Clinical studies performed with such vaccines produced by recombinant DNA technology have shown a good immunogenicity and tolerability in younger and elderly adults, the latter being important as these vaccines contain three times more HA antigens than IIVs [65,66,67,68,69,70,71,72,73].

Several different types of vaccines based on new technologies and platforms (e.g., plant-based, vector-based, VLP/NP technologies, nucleotide-based technologies, recombinant replication-deficient LAIVs) are now under development with a view to producing next-generation vaccines, in order to complement for or to overcome the drawbacks of the current technologies [46,74]. One of the most appealing approaches for next-generation vaccines is the development of a universal influenza vaccine aimed at providing robust and long-lasting protection against multiple subtypes of influenza viruses. As reported by the National Institute of Allergy and Infectious Diseases, a universal vaccine should protect against group 1 and 2 influenza viruses, should be at least 75% effective, the protection should last at least one year and should be suitable for all age groups. Ideally, the administration of a universal vaccine would avoid the need for annual reformulation and re-administration of seasonal vaccines. So far, the stem of the HA has been identified as the ideal target for antibodies induced by a universal vaccine since it remains unchanged. However, other conserved parts have been explored such as the NA, the ectodomain of the ion channel M2, and the internal viral proteins [75,76]. Another innovative approach is represented by the respiratory vaccine program launched by Moderna. The aim is to develop a combination respiratory vaccine candidate (mRNA-1230) targeting SARS-CoV-2, influenza viruses, and respiratory syncytial virus. This vaccine is designed as an annual booster in order to protect older adults against all three viruses at once and maintain immunogenicity over the years [77].

## 4. Influenza Vaccine Effectiveness

Two criteria are used to assess the performance of influenza vaccines: efficacy and effectiveness. Vaccine efficacy refers to the reduced risk of infections and subsequent disease by vaccination in ideal circumstances such as randomized placebo-controlled clinical trials. By contrast, VE refers to the reduced risk of clinical outcomes (e.g., disease incidence, hospital admission) among subjects vaccinated under “real-word” conditions and it is usually evaluated through observational studies. Although randomized placebo-controlled clinical trials are considered the “the gold standard” for measuring vaccine efficacy, they are expensive, and often pose ethical challenges. In addition, efficacy studies with influenza vaccines have a high risk of failure based on differences in attack rates and vaccine matches, resp. mismatches in different seasons. Therefore, the evaluation of VE by performing observational studies is increasingly considered to be a more appropriate approach than efficacy studies. Since 2004–2005, the most widely used method of estimating the VE of seasonal influenza vaccines has been the test-negative design (TND). In this case, patients with signs and symptoms that meet predefined clinical definitions are tested for the presence of influenza viruses; they are classified as cases if they test positive and controls if negative [78,79,80,81,82,83].

The VE of current influenza vaccines is suboptimal, having been estimated as 40% to 60% when the vaccines strains are antigenically well-matched with the circulating viruses [3,84].

VE is dependent on several factors such as the age of vaccinees, the match between the strain included in the vaccine composition and the circulating virus, egg-adaptations occurring during the production process, and the subject’s history of previous vaccination as recently supposed [3,79,82,83].

Vaccine impact is referred to as the reduction in disease incidence where some subjects have received vaccination [79,80].

### 4.1. Effectiveness in Children

Children younger than 5 years old, and especially those younger than 2 years, are at higher risk of developing serious influenza-related complications. The Centers for Disease Control and Prevention (CDC) estimates that from the 2010–2011 to the 2019–2020 influenza seasons, influenza-related hospitalizations among children younger than 5 years ranged from 7000 to 26,000 in the United States, while influenza-related deaths in children ranged from 37 to 199 from the 2004–2005 to the 2019–2020 influenza seasons [85].

Children may also play a key role in the spread of influenza infection, shedding the virus in greater quantities and for longer duration than adults. Specifically, IBVs are more likely to affect children and to cause their hospitalization or death [24,86,87].

Influenza vaccination is recommended for children aged 6 months and older; two doses at least 4 weeks apart are recommended for children aged 6–59 months at initial vaccination, while children aged 6–35 months receiving a pediatric dose [88].

Overall, it has been estimated that IIVs are able to reduce the risk of influenza in children aged 2–16 years from 30% to 11%, and LAIVs from 18% to 4% in children aged 3–16 years [89].

An overview on studies on VE conducted in children is shown in Table 2.

With regard to IIVs and children aged up to 59 months, VE varies from moderate to effective depending on the influenza season considered and, in some cases, on diagnostic sensitivity. A study performed to assess VE in preventing laboratory-confirmed influenza infection during the 2011–2012 influenza season in China, a season characterized by a considerable mismatch of H3N2 and B viruses [98], estimated a VE of 67% (95% CI: 41; 82), indicating that vaccinated children were considerably protected from developing influenza that would have required medical attention [90]. Moreover, according to the authors, no significant differences were found between children aged 6–35 months (72%, 95% CI: 43; 86) and those aged 36–59 months (50%, 95% CI: −46.3; 82.9). However, a study performed in the Republic of Korea in the influenza season 2017–2018 showed that the VE in the older age group (36–59 months) was higher than that in the younger age group (6–35 months), with 59.3% (95% CI: 8.8; 81.9) versus 44.1% (95% CI: −0.2; 67.8), respectively [91]. These numbers emphasize that it can be difficult to draw valid conclusion when comparing VE data of different seasons, resp. different regions. In addition, VE may also vary according to the diagnostic tools, such as a rapid influenza diagnostic test and the reverse transcription polymerase chain reaction (RT-PCR), the latter being considered as the gold standard for molecular detection of influenza viruses [99]. In the Republic of Korea study the adjusted VE was 53.4% (95% CI: 25.3; 70.5) according to RT-PCR and 14.8% (95% CI: −4.4; 30.0) according to the rapid influenza diagnostic test (RIDT). When the different virus subtypes were considered, the VE based on RT-PCR gave 68.8% (95% CI: 38.7; 84.1) against IAVs and 29.7% (95% CI: −35.1; 61.8) against IBV. This study was performed in the 2017–2018 season, which was characterized by a mismatch between the circulating IBV (B/Yamagata-lineage) and the vaccine strain (B/Victoria-lineage); this probably explained the low VE against IBV. This is also supported by the VE data based on the RIDT of 24.2% (95% CI: 3.1; 40.2) against influenza A and a negative VE value of −5.1% (95% CI: −42.6; 21.4) against influenza B. Another study in the US using the Department of Defense’s Data Base DoDGRS evaluated the VE against medically-attended, laboratory-confirmed influenza infections in children aged 6 months to 17 years (6–11 months; 12–23 months; 2–4 years; 5–8 years; 9–12 years and 13–17 years) over four influenza seasons (from 2016–2017 to 2019–2020) [92]. The VE against any type of influenza was low and non-significant in the youngest children (aged 6–12 months) and high in the 12–23- month age-group; it then declined linearly with increasing age with an overall VE of 42% (95% CI: 37; 47). The study also showed that the VE was also very type/subtype- and strain-specific, with the highest value of 55% (95% CI: 47; 61) against influenza A/H1N1pdm09, followed by 49% (95% CI: 41; 55) against influenza B, and being the lowest against A/H3N2 with only 37% (95% CI: 28; 45) [92]. Another study evaluating the VE among children aged 6 to 72 months during two influenza seasons (2016–2017 and 2017–2018) reported an overall VE of 58% (95% CI: 31; 74). When analyzed by age groups, 6–35 months and 36–72 months, the lowest VE (28%, 95% CI: −80; 71) was found in the youngest group [93].

Recently, it was considered that repeated vaccinations can impair the antibody response, especially in the elderly; however, this consideration is still a topic of controversial discussions [100,101,102,103,104,105,106]. The mechanism behind this phenomenon is poorly understood, however different explanations have been proposed. One is the “negative interference” or “the antigenic distance hypothesis” based on the match between the virus strains present in the prior and current vaccine and the antigenic difference with the drifted circulating virus. In this circumstance, the immune system could be too focused on the viruses contained in the vaccines and less effective against the drifted one. Another explanation may be the antibody sequestration meaning that antibodies developed following the prior vaccination bind to subsequent vaccine antigens and prevent the exposure to the immune system. Other studies have reported that repeat vaccination with the same influenza vaccines reduces antibody-affinity maturation and decreases the B-cell response [107,108,109,110,111,112,113,114]. This has been at least partially addressed in two studies [94,95]. A meta-analysis of 28 suitable published studies in children between 6 months and 17 years of age showed that influenza vaccination offered higher protection in children who were fully vaccinated (VE of 61.79%; 95% CI: 54.45; 69.13), compared to those who were partially vaccinated (33.91%; 95% CI: 21.12; 46.69) against any influenza type [94] and of 53% (95% CI: 45; 60) in 2016–2017 and 2017–2018 influenza seasons [95]. Overall, both studies [94,95] concluded that influenza vaccination provided substantial protection in children, even in seasons characterized by a high disease burden and the predominant circulation of mismatched viruses, such as H3N2 [95]. However, another study found an overall VE of 63% (95% CI: 38; 78) against critical illness due to any influenza virus, with no difference in protection between age-groups or between fully and partially vaccinated children [96]. Interestingly, this VE estimate was made during the 2019–2020 influenza season, which was characterized by a mismatch between the predominant influenza viruses and the vaccine strain; it therefore underlined the benefit of vaccination in reducing the risk of life-threatening influenza in children.

VE in subjects <17–18 years ranged from 42% (95% CI: 37; 47) [92] to 33% (95% CI: 24.3; 40.7) [97] over four consecutive influenza seasons, from 2016–2017 to 2019–2020 [92] and from 2012–2013 to 2015–2016 [97]. The consensus is that vaccination is protective in children, though age-related differences were found in some cases and the lowest protection was observed against H3N2 [92,94,95,96,97].

Overall, the question on the need of influenza vaccination in children is still open. On the one side, there is evidence of moderate IIV VE in children against medically, laboratory-confirmed influenza infection, to prevent substantial influenza associated life-threatening illness requiring invasive mechanical ventilation, a strong predictor of death, to reduce the overall risk of influenza-associated hospitalizations, and finally of a possible indirect benefit to reduce the transmission to (high-) risk groups such as elderly adults. On the other hand, VE seems to be variable and suboptimal, with differences being related to the season, child’s age, antigenic match/mismatch, and the manufacturing process. In addition, since vaccination is recommended for children aged 6 months and older, a promising strategy may be maternal immunization [86,92,95,96,97,115,116].

### 4.2. Effectiveness in Adults and the Elderly

While influenza may be serious and can affect anyone, adults aged 65 years and older have a higher risk of developing serious influenza-related complications than young and healthy adults. The CDC estimates that, in recent years, between 70% and 85% of seasonal influenza-related deaths and 50–70% of hospitalizations have occurred in subjects aged ≥65 years, defined as older adults/elderly [117,118]. Vaccination is therefore strongly recommended for the elderly, in order to prevent the disease and its related complications.

An overview on studies on VE conducted in adults and the elderly is shown in Table 3.

VE in the elderly has been evaluated in several influenza seasons from 2010–2011 to 2019–2020, and several studies have suggested that it exerts an overall protective effect even in elderly people with cardiovascular and/or respiratory diseases and those over 75 years old [119,120,124,125,130]. For instance, one study evaluated VE over eight seasons, and found that, in every season, vaccination reduced the risk of severe influenza disease in vaccinated subjects aged ≥65 years by 16% (95% CI: 12; 19) to 48% (95% CI: 41; 54) [119]. A study performed in China (Beijing) during the 2013–2014 influenza season reported that VE was favourable (59%, 95% CI: −79; 90) against H1N1, suboptimal against H3N2 (22%, 95% CI: −253; 83) and not present against B viruses (−20%, 95% CI: −239; 58); this last finding was attributed to genetic-lineage mismatch with the vaccine reference component for the B/Yamagata-lineage strain [120]. The same was observed during the 2015–2016 season and was explained by a mismatch between the circulating B-Victoria lineage and the B-Yamagata lineage viruses included in the vaccine [121].

Differences have also been observed according to the type of vaccine administered to the elderly. Adjuvanted IIVs seem to yield higher VE values than non-adjuvated IIVs, more effectively reducing the risk of hospitalization for influenza or pneumonia and significantly reducing influenza-related medical consultations [122,131]. In addition, adjuvanted IIVs are reported to significantly reduce influenza-related medical consultations in adults aged ≥65 years with at least one medical condition, in comparison with subjects receiving conventional non-adjuvanted IIVs [123]. Notably, these findings were observed during the 2017–2018 and 2018–2019 influenza seasons, which were characterized by “high” and “moderate” disease severity, respectively. The overall VE in subjects aged ≥65 years was 17% (95% CI: −14; 39) in 2017–2018 and 12% (95% CI: −31; 40) in 2018–2019 [132,133]. Moreover, the high-dose IIV also showed the potential to reduce hospitalizations and emergency room visits for influenza and pneumonia in the elderly over six influenza seasons (from 2012–2013 to 2017–2018) [134].

Further studies have compared VE between adults and the elderly, with different conclusions [124,125,126,127]. One comparison of VE over five influenza seasons (from 2011–2012 to 2015–2016) between a reference group of adults aged 18–49 years and elderly (65–74 years, ≥75 years, and ≥65 years) found an overall VE of 48% (95% CI: 41; 54) against H1N1 in the adults and of 49% (95% CI: 22; 66) in the elderly, of 55% (95% CI: 45; 63) and 62% (95% CI: 44; 74) against B viruses, and of 21% (95% CI: 9; 32) and 14% (95% CI: −14; 36) against H3N2, respectively. Excluding the 2014–2015 season, during which the circulating virus was antigenically different from the seasonal vaccine strain, the VE increased to 35% (95% CI: 22; 46) in adults and 17% (95% CI: −26; 45) in elderly. Overall, no consistent pattern of lower VE in any of the elderly subgroups compared to among adults was found, suggesting that influenza vaccination provided similar levels of protection in both the elderly and adults [125]. A further study reported a moderate VE (42.8%, 95% CI: 23.8; 57.0) against influenza-related hospitalization in subjects aged ≥16 years with a lower trend in adults aged 16–64 years (33.2%, 95% CI: −6.7; 58.2) [126]. Conversely, another two studies concluded that the protection provided by influenza vaccines was moderate against influenza-associated hospitalizations and medically-attended laboratory-confirmed infection among adults, while the VE against H3N2 was low in the elderly, especially in mismatched seasons [92,124]. The overall VE was 51% (95% CI: 44, 58) among adults, 48% (95% CI: 37; 89) against H1N1, 37% (95% CI: 24; 50) against H3N2, and 38% (95% CI: 23; 53) against B viruses, while it was significantly lower (37%) (95% CI: 30; 44) in the elderly. The biggest differences between matched and mis-matched strains were seen in persons from 65 years onwards when the VE against A(H3N2) was 43% (95% CI: 33; 53) in seasons when circulating and vaccine strains were antigenically similar and 14% (95% CI: −3; 30) when A(H3N2) variant viruses predominated which results in a negligible protection against hospitalization during seasons with mismatched H3N2 viruses [124]. A lower VE against H3N2, in addition to H1N1, in adults aged ≥65 years old was also reported in another study [127], clearly demonstrating the lower VE of IIV in this specific age-group. The reduced VE could be explained by immunosenescence, an age-related decline in the normal function of the immune system [135]. However, other factors and mechanisms may also influence the immune response to influenza vaccines and need further investigation. Moreover, influenza vaccination has been associated with a 41% reduction in the risk of hospitalization for influenza illness, with an overall VE of 41% (95% CI: 27; 52) against all influenza viruses among adults (median age 63 years) in 2019–2020 [128]. In the same season, a significant VE (53%, 95% CI: 30; 69) among working adults in Japan was reported; this contrasted with a low VE of 28% (95% CI: −5; 50) in season 2017–2018 and an even negative VE of −11% (95% CI: −42; 14) in season 2018–2019, which testifies to the fact that effectiveness of influenza vaccines may substantially vary from season to season according to the circulating influenza viruses, antigenic matches and mismatches, pre-existing immunity, and different age-groups [129].

## 5. Conclusions

It is commonly accepted in the scientific and medical communities that vaccination remains the most effective method of controlling the morbidity and mortality of seasonal influenza, especially with respect to (high-) risk groups such as young children, elderly adults, persons with chronic medical conditions, and immunocompromised persons. However, it is very difficult to evaluate the efficacy of influenza vaccines in these risk groups as placebo-controlled efficacy studies are for ethical reasons not accepted by most of the Regulatory Authorities and Ethic Committees.

So-called effectiveness studies which investigate the positive effects of influenza vaccines in the field are an excellent alternative. However, the results of such effectiveness studies are very divergent, and results are sometimes even contradictive. Nevertheless, the selection of single studies or meta-analyses of a variety of studies confirm that in general influenza vaccines are effective against morbidity and mortality in all age and risk groups, especially in young children and elderly adults. However, the outcome of such effectiveness studies is very dependent on influenza virus-specific parameters, mainly the antigenic drift, especially of the A-strains or the differently circulating B-strains of the two divergent lineages B-Victoria and B-Yamagata. The negative impact of the latter was overcome by the introduction of the quadrivalent vaccines with representative vaccine strains of both lineages. A more challenging issue is the antigenic drift of the A strains, especially for H3N2 viruses, which can result in a complete lack of protection in specific seasons. This issue can be exacerbated by the conventional influenza vaccine production technology of embryonated hens’ eggs which carries the risk of antigenically mis-matched vaccine virus by the introduction of egg-adapted mutations. This issue can be overcome by new technologies such as cell culture or the use of recombinant vaccines which ensure the antigenic similarity of the vaccine strains with the wildtype viruses circulating in nature. Nevertheless, even these vaccines may face the limitations of potential mismatches between the circulating strains and the WHO-recommended vaccine strains caused by antigenic drift, especially during an influenza season. However, novel technologies, namely recombinant RNA/DNA-platforms, may offer a higher flexibility in addressing antigenic changes during an influenza season potentially allowing the adaptation of the vaccine during the season. This, of course, would also require higher flexibility and accelerated responses of Regulatory Authorities.

Finally, the experience based on a variety of effectiveness studies would strongly raise the bar for so-called “universal influenza vaccines” or, more precise, “influenza vaccines with a broadened and longer-lasting immunity” would require studies over several seasons without any prediction of the importance of the antigenic drift over these seasons carrying a high risk of failure within one specific season. Therefore, such studies most likely cannot be performed by individual vaccine producers but may need the intensive support of all stakeholders involved in the timely production of seasonal influenza vaccines, i.e., the WHO, International Institutions, the scientific community, and, last, but not least, Regulatory Authorities worldwide.

## Figures and Tables

**Table 1:** Summary of advantages and disadvantages of licensed seasonal influenza vaccines.

Licensed Vaccines	Advantages	Disadvantages
Inactivated egg-based	✓ Extensive safety data available ✓ Cost-effectiveness ✓ High yields of influenza antigens	Huge number of eggs Theoretical risk of anaphylactic reaction Poor growth for some viruses (i.e., H3N2) Egg-adaptation
Inactivated cell-based	✓ Independence from eggs supply ✓ Free from egg components and adaptation	Shorter experience Need of qualified production facilities Higher production costs Extended quality control program
LAIV	✓ Administration route ✓ Broader humoral and cellular responses ✓ Protection against both well-matched and non-matching influenza strains	Not recommended for immunocompromised subjects
Recombinant HA	✓ Independence from eggs supply ✓ Viral RNA sequence to start the process	Additional studies are needed

**Table 2:** Overview of studies on VE conducted in children.

Age	Country/ Continent	Season(s)	VE (95% CI)	Reference
6–59 months	China	2011–2012	67% (41–82) 72% (43–86) in 6–35 months 50% (−46.3–82.9) in 36–59 months	Wang, Y. et al., 2016 [90]
6–59 months	Republic of Korea	2017–2018	53.4% (25.3–70.5) 44.1% (−0.2–67.8) in 6 months–2 years 59.3% (8.8–81.9) in 3–5 years	Sohn, Y.J. et al., 2020 [91]
6 months–17 years	US	From 2016–2017 to 2019–2020	42% (37–47) 46% (29–59) in 6–23 months, 50% (39–59) in 2–4 years; 44% (34–53) in 5–8 years, 37% (21–50) in 9–12 years, 34% (20–46) in 13–17 years	Hu, W. et al., 2021 [92]
6–72 months	China	2016–2017 and 2017–2018	58% (31–74) 68% (41–82) in 36–72 months, 28% (80–71) in 6–35 months	Luo, S.Y. et al., 2019 [93]
6 months–17 years	Asia, Australasia, Europe, North America, South America	From 2005–2006 to 2018–2019	57.48% (49.46–65.49) 61.71% (49.29–74.12) in ≤5 years, 54.37% (35.14–73.60) in 6–17 years	Kalligeros, M. et al., 2020 [94]
<18 years	US	2016–2017 and 2017–2018	49% (42–56) 48% (40–55) in 6 months–8 years, 42% (21–58) in 9–17 years	Kim, S.S. et al., 2021 [95]
<18 years	US	2019–2020	63% (38–78) 66% (35–82) in 6 months–8 years, 62% (−16–88) in 9–17 years	Olson, S.M. et al., 2022 [96]
2–17 years	Germany	From 2012–2013 to 2015–2016	33% (24.3–40.7)	Mohl, A. et al., 2018 [97]

**Table 3:** Overview of studies on VE conducted in adults and the elderly.

Age	Country/ Continent	Season(s)	VE (95% CI)	Reference
65–100 years	Finland	From 2012–2013 to 2019–2020	From 16% (12–19) to 48% (41–54)	Baum, U. et al., 2021 [119]
≥60 years	China	2013–2014	32% (−48–69)	Zhang, L. et al., 2018 [120]
≥65 years	Denmark	2015–2016	35.0% (11.1–52.4) against H1N1, 4.1% (22.0–24.7) against B	Emborg, H.D. et al., 2016 [121]
≥65 years	US	2017–2018 and 2018–2019	From 7.7% (2.3–12.8) to 18.2% (15.8–20.5) in 2017–2018, from 6.9% (3.1–10.6) to 27.8% (25.7–29.9) in 2018–2019	Boikos, C. et al., 2021 [122]
≥65 years	US	2017–2018 and 2018–2019	From −0.8% (−8.9–6.6) to 7.1% (3.3–10.8)	Boikos, C. et al., 2021 [123]
≥18 years	Europe, North America, Oceania, Asia	From 2010–2011 to 2014–2015	41% (34–48) 51% (44–58) in 18–64 years, 37% (30–44) in ≥65 years	Rondy, M. et al., 2017 [124]
≥18 years	US	From 2011–2012 to 2015–2016	48% (41–54) in adults and 49% (22–66) in the elderly against H1N1, 55% (45–63) and 62% (44–74) against B, 21% (9–32) and 14% (−14–36) against H3N2	Russell, K. et al., 2018 [125]
≥16 years	Canada	2011–2012	42.8% (23.8–57.0) in ≥16 years 33.2% (−6.7–58.2) in 16–64 years	Andrew, M.K. et al., 2017 [126]
≥18 years	US	From 2016–2017 to 2019–2020	40% (33–46) 44% (35–52) in 18–49 years, 41% (29–51) in 50–64 years, 28% (0–49) in ≥65 years	Hu, W. et al., 2021 [127]
≥18 years	US	2019–2020	41% (27–52) 16% (−28–44) in 18–49 years, 40% (16–57) in 50–64 years, 54% (35–67) in ≥65 years	Tenforde, M.W. et al., 2021 [128]
18–64 years	Japan	From 2017–2018 to 2019–2020	From −11% (−42–14) to 53% (30–69)	Tadakuma, K. et al., 2022 [129]

## Data Availability

Not applicable.
